# Impact of intubated vs. non-intubated anesthesia on postoperative diaphragmatic function: Results from a prospective observational study

**DOI:** 10.3389/fphys.2022.953951

**Published:** 2022-08-08

**Authors:** Irene Steinberg, Agnese Bisciaio, Giulio Luca Rosboch, Edoardo Ceraolo, Francesco Guerrera, Enrico Ruffini, Luca Brazzi

**Affiliations:** ^1^ Department of Surgical Sciences, University of Turin, Turin, Italy; ^2^ Department of Anaesthesia, Intensive Care and Emergency—‘Città Della Salute e Della Scienza University Hospital, Turin, Italy; ^3^ Department of Thoracic Surgery—‘Città Della Salute e Della Scienza University Hospital, Turin, Italy

**Keywords:** diaphragmatic function, thoracic surgery, general anesthesia, non-intubated anesthesia, diaphragmatic ultrasound

## Abstract

**Background:** An altered diaphragmatic function was associated with the development of postoperative pulmonary complications following thoracic surgery.

**Methods:** To evaluate the impact of different anesthetic techniques on postoperative diaphragmatic dysfunction, patients undergoing video-assisted thoracoscopic surgery (VATS) lung biopsy for interstitial lung disease were enrolled in a monocentric observational prospective study. Patients received intubated or non-intubated anesthesia according to risk assessment and preferences following multidisciplinary discussion. Ultrasound measured diaphragmatic excursion (DIA) and Thickening Fraction (TF) were recorded together with arterial blood gases and pulmonary function tests (PFT) immediately before and 12 h after surgery. Pain control and postoperative nausea and vomiting (PONV) were also evaluated.

**Results:** From February 2019 to September 2020, 41 consecutive patients were enrolled. Five were lost due to difficulties in collecting postoperative data. Of the remaining 36 patients, 25 underwent surgery with a non-intubated anesthesia approach whereas 11 underwent intubated general anesthesia. The two groups had similar baseline characteristics. On the operated side, DIA and TF showed a lower residual postoperative function in the intubated group compared to the non-intubated group (54 vs. 82% of DIA and 36 vs. 97% of TF; *p* = 0.001 for both). The same was observed on the non-operated side (58 vs. 82% and 62 vs. 94%; *p* = 0.005 and *p* = 0.045, respectively, for DIA and TF). No differences were observed between groups in terms of pain control, PONV, gas exchange and PFT.

**Conclusion:** This study suggests that maintenance of spontaneous breathing during VATS lung biopsy is associated with better diaphragmatic residual function after surgery.

## Introduction

The effect of anesthesia and muscle-relaxing agents on diaphragmatic function has been first described by Froese et al., in 1974 (Froese and Bryan, 1974). In healthy volunteers, they found a significant displacement of the diaphragm and a reduction in functional residual capacity (FRC). Hedenstierna et al. later confirmed these results (Hedenstierna et al., 1985).

The impact of different anesthetics on the diaphragm has been investigated in both clinical and preclinical settings. Anesthetic gas, phenobarbital, propofol and opiates have been shown to impair diaphragmatic function (Sasaki et al., 2013). A similar effect has been attributed to mechanical ventilation (Gayan-Ramirez et al., 2003). However, these alterations were observed during anesthesia, without investigating whether they persisted afterward. Moreover, studies investigating diaphragmatic function after surgery found a substantial alteration in its excitability patterns (Berdah et al., 2002) with a persistent reduction in FRC (Meyers, 1975) despite optimization of pain control (Simonneau et al., 1983). Therefore is difficult to understand the relative role of anesthesia, mechanical ventilation, surgery and its related inflammation (Krause et al., 1998) in causing diaphragmatic dysfunction.

Also, a recent study by Spadaro et al. found an association between diaphragmatic dysfunction and postoperative pulmonary complications in thoracic surgery (Spadaro et al., 2019). They measured the diaphragmatic function of patients who underwent thoracotomy or video-assisted thoracoscopic surgery (VATS) and found that VATS patients had a lower reduction in diaphragmatic excursion and fewer pulmonary complications.

Non-intubated thoracic surgery is emerging as a feasible and safe approach for a minimally invasive procedure, such as atypical resections for pulmonary biopsy (Pompeo et al., 2013). Especially in the context of interstitial lung disease where patients present with severely altered baseline pulmonary function test (Pompeo et al., 2019). Anyhow, even though evidence in favor of this approach is emerging (Guerrera et al., 2021), there is still no definitive confirmation of the superiority of this approach in terms of postoperative complications and outcomes.

Lung biopsy surgery, which in our center is performed with both intubated and non-intubated anesthesia, seemed the optimal setting to evaluate the impact of general anesthesia alone on diaphragmatic function. Our primary objective was to compare ultrasonographic parameters of residual postoperative diaphragm function after VATS lung biopsy in intubated and non-intubated patients.

## Materials and methods

Patients undergoing lung biopsy surgery for the diagnosis of interstitial lung disease were enrolled in a prospective observational study to evaluate: 1) postoperative diaphragmatic function (primary endpoint); 2) achievement of pain control and occurrence of postoperative nausea and vomiting (PONV) after intubated or non-intubated anesthesia (secondary endpoint). The study was approved by the local ethical committee (Comitato Etico Interaziendale A.O.U.Città della Salute e della Scienza di Torino/A.O.Ordine Mauriziano/ASL Città di Torino, protocol n.0000060, January 2nd, 2019) and written informed consent was obtained from all patients. Primary and secondary outcomes were defined and established *a priori* at the initiation of the study as well as group allocation according to anesthetic management. This manuscript adheres to the applicable EQUATOR guidelines.

### Study population

Patients were enrolled from February 2019 to September 2020. Exclusion criteria were age <18, pregnancy, Body Mass Index >35, inability to provide informed consent, American Society of Anesthesiologists physical status classification score of IV or recommended postoperative ICU admission.

In the absence of clear evidence about the advantages and disadvantages of intubation in the context of video-assisted thoracoscopic surgery (VATS) lung biopsy, the decision on the anesthetic technique to be adopted in the individual case was agreed upon between patients and the operator according to clinical conditions, preferences and the specific risks associated with the procedure.

### Measurements

**FIGURE 1:**
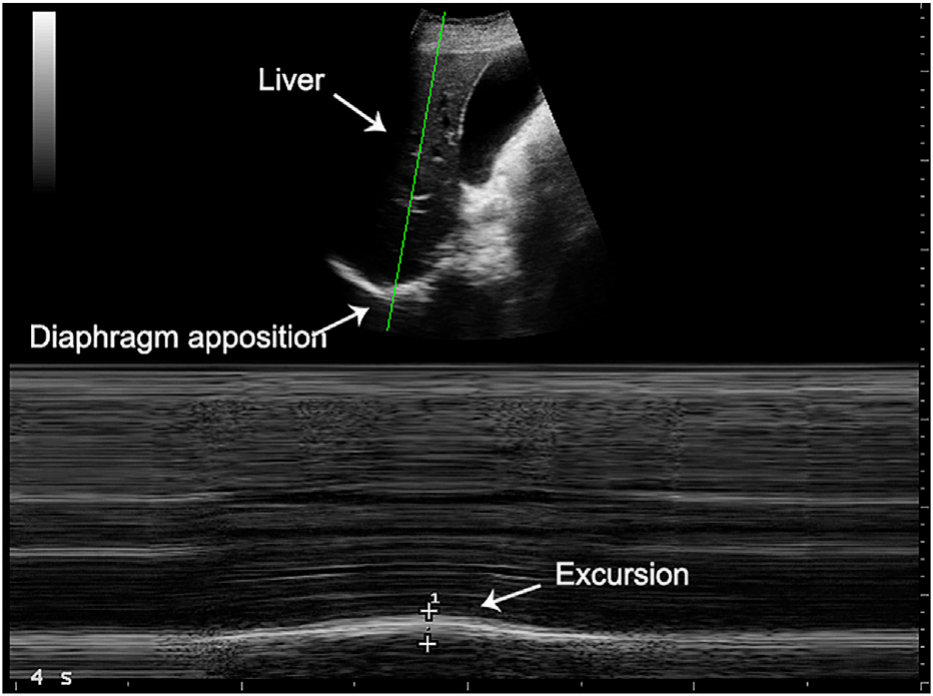
Diaphragmatic excursion measurement.

Measurements were performed the day before and 12 hours after surgery. At enrollment, patient history and baseline blood gas analysis (ABG) were collected. Pulmonary function test (PFT), forced vital capacity (FVC) and forced expiratory volume in the first second (FEV1) were performed with the portable Spirobank II^®^ Smart system (MIR—Rome, Italy) according to the American Thoracic Society technical statement (Culver et al., 2017) and expressed as the percentage of the predicted value (Laveneziana et al., 2019). A trained anesthetist performed an ultrasound assessment of the diaphragm in the semi-recumbent position with the bed angulated at 45° using a MyLab 25 device (Esaote—Genoa, Italy). The assessment was performed on both the operated and non-operated side of the thorax. Thickening fraction and diaphragmatic excursion were recorded during a deep breath, starting from FRC to maximum inspiratory reserve, to investigate diaphragmatic maximal force and increase sensibility in detecting a postoperative change in function. The diaphragmatic excursion was measured with a low frequency (3.5–5 MHz) convex probe placed below the right and left costal margin, between mid and anterior axillary line and directed dorsally, medially and cranially to intersect the posterior part of the diaphragm with an angle as close as possible to 90°. When correct visualization was obtained, the ultrasound machine was switched to M-Mode and the patient was asked to breathe deeply. The image was frozen and the distance between the expiratory and inspiratory position of the diaphragm was measured to calculate excursion (DIA) (Boussuges et al., 2009) (Figure 1).

**FIGURE 2:**
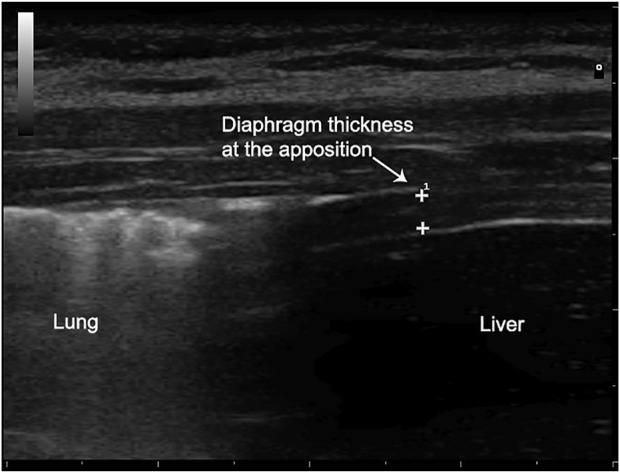
Diaphragmatic thickness measurement.

Diaphragm thickness in the zone of apposition to the rib cage was measured using a high frequency (10 MHz) linear probe. The probe was placed around the 8th to the 11th intercostal space between the mid and anterior axillary line in a craniocaudal direction. Parietal pleura and peritoneum were visualized as two parallel white rows demarcating the diaphragm thickness, which was measured in both deep breathing and end-expiration, and then computed as the percentage of Thickening Fraction (TF) = (End Inspiratory thickness-End Expiratory thickness)/End Expiratory thickness (Wait et al., 1989), (Tuinman et al., 2020) (Figure 2).

Twelve hours after surgery, data on anesthesia type and gas exchange were recorded. Numeric rating scale (NRS), both at rest and during deep breathing, was measured before PFT and ultrasound assessment. Due to the clinical setting, it was not possible to blind ultrasound operators to the type of anesthesia received by the patients.

### Anesthetic management and surgical procedure

Anesthesia was conducted according to local protocols by the same anesthetic team. Surgical technique was the same in non-intubated and intubated patients as described by Pompeo et al. (Pompeo et al., 2013).

### Anesthetic management of non-intubated patients

In non-intubated patients, an epidural catheter was placed at T5-T6, following current best practice recommendations (Author Anonymous, 2017), and the correct position was assessed with 60 mg of 2% lidocaine. An anesthetic bolus of 0.5 mg/kg of ropivacaine was administered to complete anesthesia of the thoracic wall. To reduce chough reflex, and hence possible movements of the patient during the procedure, lidocaine aerosol was used (5 ml of 2% lidocaine with 5 ml of saline) (Trochtenberg, 1994). Proper anesthesia level was checked before skin incision and no adjunctive local anesthetic was needed in any patients. The cumulative dose of anesthetics drugs never exceeded the dosage recommended by the manufacturer.

To improve patient comfort through the procedure, mild sedation with a continuous infusion of propofol (2–4 mg kg^−1^·h^−1^) and remifentanil (0, 02-0,1 γ·kg^−1^ min^−1^) was administered. Spontaneous breathing was maintained through the whole procedure, supplementary oxygen was delivered via face mask according to patients’ necessity and etCO2 monitoring. Postoperative analgesia was managed with paracetamol plus an elastomeric pump with 0.2 mg/ml ropivacaine, usually set between 3 and 5 ml/h, and ketorolac as rescue therapy.

### Anesthetic management of intubated patients

In intubated patients, in the absence of a locoregional analgesia gold standard approach at the time the study was conducted (Steinthorsdottir et al., 2014), epidural or an interfascial plane block, such as serratus anterior or erector spinae (Gaballah et al., 2019), were performed preoperatively based on operator assessment.

Anesthesia was induced with Propofol plus opiates (usually remifentanil) and muscle paralysis was achieved with Rocuronium. Patients were intubated with an appropriately sized double-lumen left tube and connected to a Drager Zeus ventilator (Drägerwerk AG & Co. KGaA, Lübeck, Germany). Mechanical ventilation was set on Ideal Body Weight to remain between 6 and 8 ml/kg in bipulmonary ventilation and 6 ml/kg in one-lung ventilation (Kilpatrick and Slinger, 2010). Anesthesia was maintained with Desflurane. At the end of the procedure, muscle paralysis was fully reversed by Sugammadex (Bridion ^®^) dosed according to the level of neuromuscular block evaluated by train-of-four (TOF) scan. Patients were extubated when a TOF ratio >95% was achieved. Postoperative analgesia was managed with paracetamol plus tramadol, and ketorolac as rescue therapy.

### Statistical method

Statistical analysis was conducted with R (R Foundation for Statistical Computing version 4.0). The comparison between baseline and postoperative function was performed by computing the percentage of residual function according to the formula:

$$\mathrm{Residual}\;\mathrm{function}=\;\frac{\mathrm{postoperatiove}\;\mathrm{value}}{\mathrm{preoperative}\;\mathrm{value}}*100$$ (1)

The normal distribution of data was tested by the Shapiro–Wilk Normality Test. Normally distributed continuous variables are described as mean and standard deviation and evaluated by *t*-test for independent samples whereas non-normally distributed variables are described as median and interquartile range and evaluated by non-parametrical Mann-Whitney’s *U* test for independent samples. Pearson’s chi-square test or Fisher’s exact test were used to compare categorical data. P < 0.05 was considered statistically significant. Two-way Analysis of Variance (ANOVA) comparison for repeated measure, after checking necessary assumptions were met, was used to compare differences with time. A multiple linear regression model was constructed to check the impact of confounding variables.

### Sample size

The sample size was calculated by considering the primary endpoint: the reduction of the maximum diaphragmatic function in patients receiving intubation compared to the non-intubated patient. Welvaart et al. (Welvaart et al., 2011) analyzing the contractility of the muscle fibers of the diaphragm both at baseline and after 2 h of anesthesia for thoracic surgery observed a 35% reduction in the capacity to generate force. Assuming that surgery without intubation would not affect the diaphragmatic function and considering an enrollment ratio of 2:1 based on usual practice at our institution, it was estimated that a minimum of 24 patients (16 non-intubated and 8 intubated patients) needed to be enrolled to reach 90% potency with an alpha of 0.05.

### Missing data

Missing data due to technical difficulties account for less than 5% of key variables except for PFT (25% missing values). Due to the COVID-19 outbreak in northern Italy, enrollment was temporary on hold in March 2020. After March 2020 nine patients were enrolled, despite all patients tested negative for SarsCov-2 before surgery, to reduce the possibility of patient and operator exposure they did not receive adjunctive PFT. Missing data were handled with pairwise deletion of the observations.

## Results

Forty-one consecutive patients were enrolled, five were excluded due to the inability to appropriately collect all postoperative data. Baseline characteristics of excluded patients were not statistically different (Supplementary Table S1). Of the remaining thirty-six patients, twenty-five underwent non-intubated anesthesia, whereas eleven received intubated anesthesia. No differences were found between the two groups in baseline characteristics that could act as potential confounders (Table I). Binary logistic regression models were built to detect a possible effect of baseline patients’ characteristics on the anesthetic approach. Neither age, BMI, gender, gas exchange, PFT nor comorbidities were associated with intubation.

**TABLE 1:** Baseline characteristics.

	Overall (36)	Non-intubated (25)	Intubated (11)	p
Gender male/female	20/16	15/10	5/6	0.48
Age	60 (±15)	60 (±16)	59 (±13)	0.77
Body Mass Index	24.7 (±3.6)	25.3 (±3.3)	23.2 (±3.9)	0.13
Comorbidities Arterial hypertension	13	10	3	0.71
Previous myocardial infarction	2	2	0	1.00
Congestive heart failure	1	1	0	1.00
Chronic obstructive pulmonary disease	2	2	0	1.00
Pulmonary fibrosis	1	1	0	1.00
Diabetes mellitus	5	4	1	1.00
Previous stroke	3	1	2	0.22
Chronic kidney disease	1	1	0	1.00
Hepatic failure	1	1	0	1.00
Surgical site right/left	27/9	20/5	7/4	0.41
Baseline paO2	76 [68 to 91] mmHg	75 [66 to 85] mmHg	82 ([70 to 98] mmHg	0.29
Baseline paCO2	37 [35 to 42] mmHg	38 [35 to 44] mmHg	37 [36 to 40] mmHg	0.66
Baseline FVC%^a^	64 (±22)	62 (±22)	71 (±21)	0.37
Baseline FEV1%^b^	64 (±25)	62 (±26)	70 (±23)	0.48
Baseline FEV1/FVC%^c^	90 [62 to 120]	87 [63 to 119]	100 [62 to 124]	0.87
Baseline maximal DIA^d^ on operated side	4.2 (±1.4) mm	3.9 (±1.4) mm	4.7 (±1.2) mm	0.11
Baseline maximal DIA^d^ on non-operated side	4.0 (±1.3) mm	3.8 (±1.4) mm	4.3 (±1.2) mm	0.33
Baseline maximal TF^e^ on operated side	53% [38 to 71]	50% [33 to 69]	59% [41 to 72]	0.46
Baseline maximal TF^e^ on non-operated side	59% [40 to 75]	58% [40 to 75]	67% [43 to 89]	0.54

a FVC%, forced vital capacity percentage of predicted.

b FEV1%, forced expiratory volume in the first second percentage of predicted.

c FEV1/FVC% percentage of predicted ratio between FEV1 and FVC.

d DIA, diaphragmatic excursion.

e TF, thickening fraction.

Normally distributed variables are reported as mean (±SD) and non-normally distributed variables as median [IQR]. Fisher exact test was used for dichotomous variables, t-student test for normally distributed continuous variables and non-parametric Mann-Whitney test for continuous variables not normally distributed.

### Primary outcome

**FIGURE 3:**
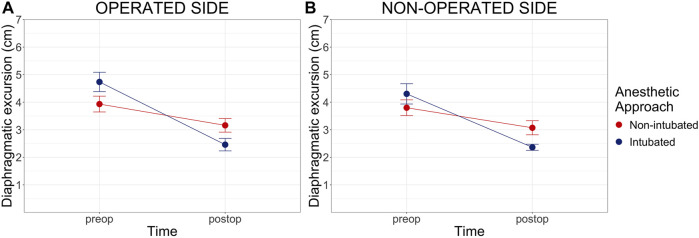
Preoperative and Postoperative maximal diaphragmatic excursion. Preop = preoperative, postop = postoperative. Cm of diaphragmatic excursion pre and post-operatively on both the operated (Panel A) and non-operated side (Panel B). Diaphragmatic excursion data are shown as mean and standard error (error bars). Two-way analysis of variance (ANOVA) comparison for repeated measure between groups: *p* = 0.001 on the operated side and *p* = 0.005 on the non-operated side.

In the non-intubated group the preoperative maximal diaphragmatic excursion was 3.93 cm (CI 3.34-4.52; SD = 1.43) on the operated side and 3.80 cm (CI 3.31-4.39; SD = 1.4) on the non-operated side. Post-operative excursion was 3.16 cm (CI 2.65-3.67; SD = 1.24) and 3.07 cm (CI 2.54-3.60; SD = 1.25), respectively. In the intubated group diaphragmatic excursion decreased from 4.74 cm (CI 3.95-5.12; SD = 1.16) to 2.46 cm (CI 1.45-2.96; SD = 0.75) on the operated side and from 4.3 cm (CI 3.47-5.14; SD = 1.17) to 2.51 cm (CI 2.11-2.92; SD = 0.60) on the non-operated side (Figure 3). A statistically significant difference in preoperative vs. postoperative diaphragmatic excursion was found between the two groups (*p* = 0.001 and *p* = 0.005 for the operated and non-operated sides, respectively). Individual values of preoperative and postoperative diaphragmatic excursions on both sides are displayed in Supplementary Figure S1.

As shown in Table II, the residual postoperative diaphragmatic excursion was 82% on both sides in the non-intubated group and 54 and 58% on the operated and non-operated side in the intubated group (*p* = 0.001 and *p* = 0.005, respectively). We found a residual TF, of 97% on the operated side and 94% on the non-operated side in the non-intubated group, whereas intubated patients showed a residual function of 36% on the operated side and 62% on the non-operated side (*p* = 0.001 and *p* = 0.045, respectively). Gas exchange and PFT residual function were not statistically different between the two groups. Entering the anesthesia type and patients’ baseline characteristics (age, BMI, gender, gas exchange and PFT) in multivariable linear regression models, the anesthetic approach was the only variable that reached statistical significance (*p* < 0.05) in relation to diaphragmatic residual function.

**TABLE 2:** Postoperative residual function of key variables.

	Non-intubated (25)	Intubated (11)	p
P/F^a^	90% (±18)	83% (±10)	0.89
pCO2	99% [93 to 105]	93% [92 to 119]	0.96
FVC%^b^	58% (±17)	54% (±30)	0.64
FEV1%^c^	61% (±21)	60% (±38)	0.77
FEV1/FVC%^d^	81% (±36)	72% (±44)	0.38
DIA^e^ operated side	82% (±22)	54% (±19)	0.001
DIA^e^ non-operated side	82% (±23)	58% (±16)	0.005
TF^f^ operated side	97% [73 to 144]	36% [30 to 56]	0.001
TF^f^ non-operated side	94% [68 to 187]	62% [50 to 93]	0.045

a P/F, PaO2/FiO2.

b FVC%, forced vital capacity percentage of predicted.

c FEV1%, forced expiratory volume in the first second percentage of predicted.

d FEV1/FVC% percentage of predicted ratio between FEV1 and FVC.

e DIA, diaphragmatic excursion.

f TF, thickening fraction.

Normally distributed variables are reported as mean (±SD) and non-normally distributed variables as median and [IQR]. t-student test for normally distributed continuous variables and non-parametric Mann-Whitney test for continuous variables not normally distributed.

### Secondary outcomes

For pain control, all patients in the non-intubated group received epidural while in the intubated group two patients received epidural, two interfascial plane blocks (one serratus anterior and one erector spinae) and seven were managed with intravenous analgesic drugs. The median resting NRS was 1 (IQR 0–4) in the non-intubated group and 3 (IQR 2–6) in the intubated group (*p* = 0.058). During deep breathing, NRS median value was 5 in both groups (*p* = 0.145; IQR 2 to 6 in the non-intubated and 5 to 8 in intubated patients).

The association between NRS in deep breathing and residual diaphragmatic function was investigated through multiple linear regression models based on anesthesia type and NRS value. NRS was not associated with a diaphragmatic decrease in function as detected by DIA nor TF (*p* = 0.29 and *p* = 0.18, respectively, for the operated side, Supplementary Figure S2; and *p* = 0.20 and *p* = 0.38 respectively for the non-operated side, Supplementary Figure S3). Intubated anesthesia was associated with a reduction in residual postoperative diaphragmatic function in all four models (*p* < 0.05). No statistically significant difference was found between groups in the incidence of PONV (*p* = 0.297).

## Discussion

The main results of this study are 1) Intubated lung biopsy surgery is associated with a reduced residual diaphragmatic function after surgery; 2) The anesthetic approach is a stronger predictor of a reduced diaphragmatic residual function after surgery than the efficacy of pain control.

In recent years, the diaphragm and its role in maintaining respiratory function have been the subject of several studies (Supinski et al., 2018). Diaphragmatic dysfunction was associated with difficult weaning from mechanical ventilation and mortality (Jung et al., 2016). It could be argued that this concern marginally the peri-operative context since the duration of anesthesia and mechanical ventilation is generally limited. However, there is experimental evidence reporting the early impact of anesthesia and mechanical ventilation on diaphragmatic function. Powers et al. found a significant reduction in the maximum diaphragmatic force in rats subjected to 12 h of mechanical ventilation (Powers et al., 2002). Gayan-Ramirez et al. highlighted how the force generated by the diaphragm is reduced in anesthetized rats, regardless of the maintenance of spontaneous breathing (Gayan-Ramirez et al., 2003).

In humans, studying diaphragm samples obtained after the induction of anesthesia and after 2 hours from the start of thoracic surgery, Welvaart et al. found a reduction in force-generating capacity of muscle fibers of 35% (Welvaart et al., 2011). These results seem to suggest that even short general anesthesia may alter diaphragmatic function.

A reduced postoperative diaphragmatic excursion has been observed both in thoracic (Spadaro et al., 2019) and general surgery (Kim et al., 2010). Anyhow, in these studies, it was not possible to know whether anesthesia or surgery were responsible for the reduction of the diaphragmatic function.

In our study, a group of patients did not receive general anesthesia and we were able to observe the relative effect of the two components. We found a statistically significant difference between the two groups in both residual diaphragmatic excursion and thickening fraction. These results were associated only with the anesthetic approach at multiple linear regression.

The reduction we found in postoperative diaphragmatic function did not impact gas exchange and PFT. Consequently, it could be argued that this difference may not be relevant for the outcome. It should be noted, however, that the sample size of our study was not powered to detect a difference in postoperative pulmonary complications. Because Spadaro et al. found that a reduced postoperative diaphragmatic function was associated with postoperative pulmonary complications (Spadaro et al., 2019), in the context of VATS for pulmonary biopsy, a non-intubated anesthesia approach may be associated with a more favorable outcome.

Due to the different anesthetic approaches, all non-intubated patients received postoperative epidural analgesia (the catheter was necessary to allow surgical anesthesia), while, in the intubated group, epidural analgesia was deemed necessary only in two cases. Consequently, to evaluate if the difference in residual diaphragmatic function could be associated with factors other than the anesthetic approach, we investigated the association with the reported pain level. Anyhow, the constructed multiple regression models found the anesthetic approach to be a better predictor of reduced diaphragmatic function as compared to pain level.

Our results are in accordance with previous studies that did not find a correlation between diaphragmatic loss of function and the achievement of adequate pain control (Simonneau et al., 1983). In the context of upper abdominal surgery, it has been shown that epidural analgesia seems to have a positive effect on diaphragmatic dysfunction, reducing its loss in excursion (Pansard et al., 1993), while the only study that investigated the effect of epidural on diaphragmatic dysfunction in thoracic surgery found no significant benefit (Fratacci et al., 1993). A possible explanation for this difference is provided by a recent animal model. The authors linked the loss of diaphragmatic function in upper abdominal surgery to a decrease in phrenic motor output generated by peripheral afferent nerves from the abdominal wall (Chae et al., 2018).

We are aware that our study has limitations such as its observational nature and therefore the lack of randomization and perfectly homogeneous anesthetic management. Due to the clinical setting, it was impossible to blind ultrasound operators to patient groups. Moreover, the reproducibility of the voluntary respiratory maneuvers and the lack of a skin marker might have affected our results. Anyhow, it provides new insights into the mechanisms of postoperative diaphragmatic dysfunction and indicates that maintenance of spontaneous breathing during VATS lung biopsy is associated with better diaphragmatic residual function after surgery.

## Data Availability

The raw data supporting the conclusions of this article will be made available by the authors, without undue reservation.
