# Supplementary material for: Impact of intubated vs. non-intubated anesthesia on postoperative diaphragmatic function: Results from a prospective observational study
Source: Front Physiol. 2022 Aug 8;13:953951. doi: 10.3389/fphys.2022.953951 (PMC9393254; doi:10.3389/fphys.2022.953951)
Supplement: Supplementary file 1 [file DataSheet1.docx]

Supplementary Figure 1


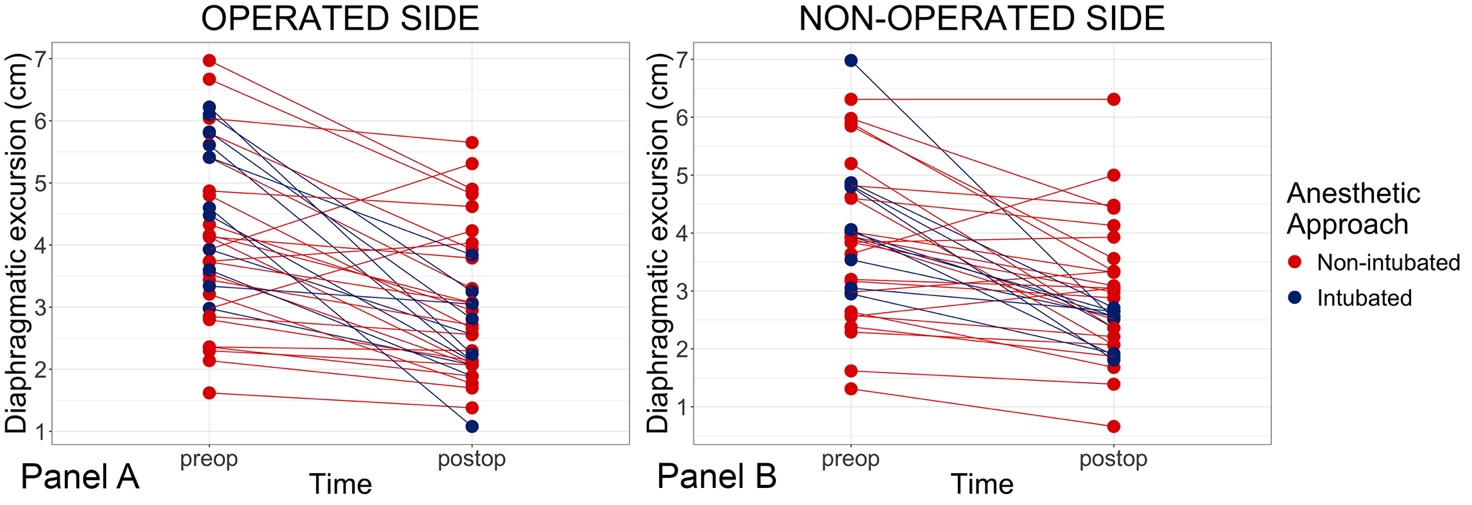


Preop=preoperative, postop=postoperative. Cm of diaphragmatic excursion pre and post operatively on both the operated (Panel A) and non-operated side (Panel B). Diaphragmatic excursion data are shown for each patient.

Supplementary Figure 2

~~
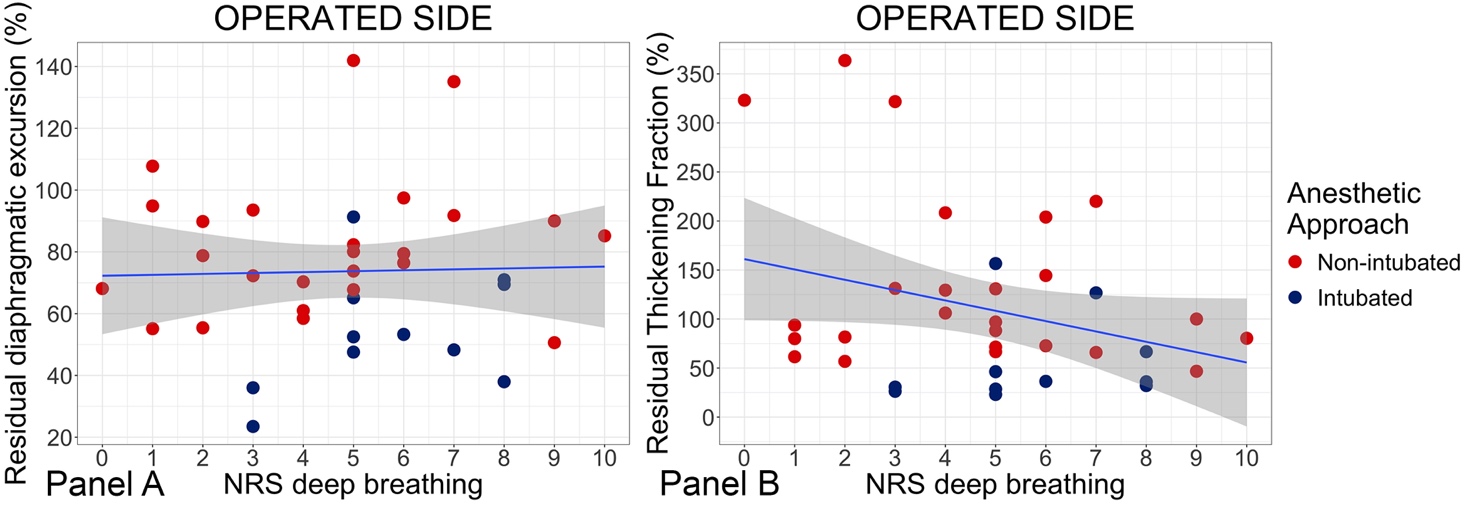
~~

NRS = numeric rating scale. Multiple regression model for residual diaphragmatic excursion (panel A) and residual thickening fraction (panel B) based on the anesthetic approach and the NRS in deep breathing for the operated side. Panel A p=0.29 for NRS and p<0.001 for anesthetic approach, Panel B p=0.18 for NRS and p=0.02 for anesthetic approach.

Supplementary Figure 3


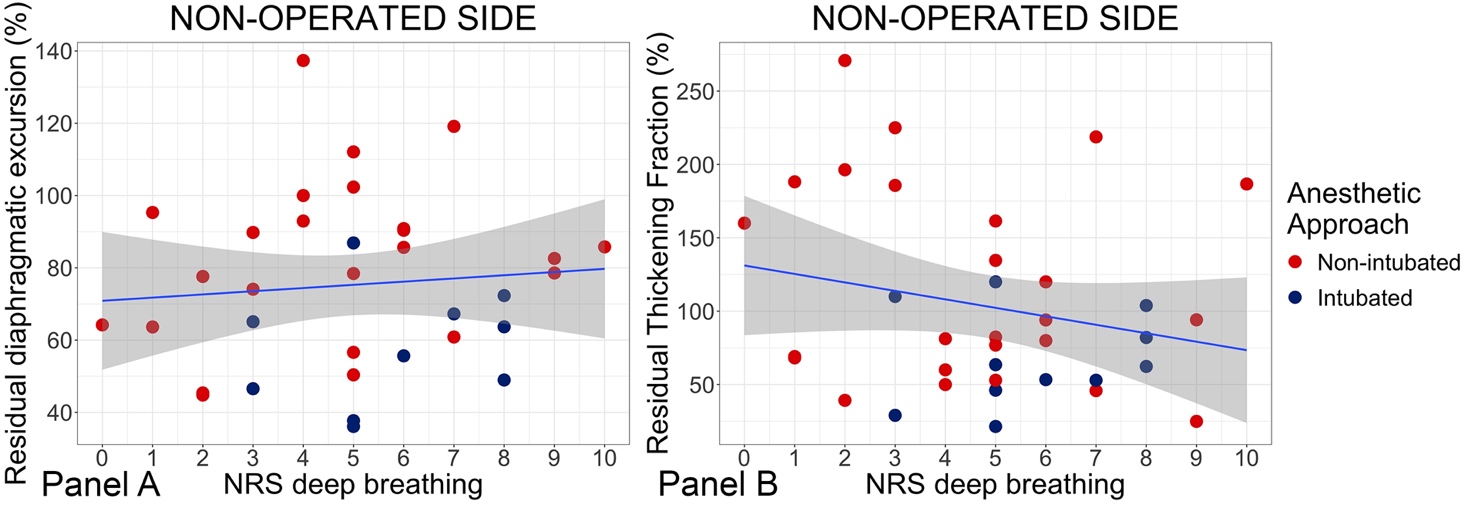


NRS = numeric rating scale. Multiple regression model for residual diaphragmatic excursion (panel A) and residual thickening fraction (panel B) based on the anesthetic approach and the NRS in deep breathing for the non-operated side. Panel A p=0.20 for NRS and p=0.002 for anesthetic approach, Panel B p=0.38 for NRS and p<0.05 for anesthetic approach.

Supplemental Table I. Baseline characteristics of excluded patients

|  | Included (36) | Excluded (5) | p |
| --- | --- | --- | --- |
| Gender  Male/Female | 20/16 | 2/3 | 1.0 |
| Age | 60 (SD ±15) | 62 (SD ±16) | 0.75 |
| Body Mass Index | 24.7 (SD ±3.6) | 26.3 (SD ±3.0) | 0.35 |
| Comorbidities  Arterial Hypertension  Previous myocardial infarction  Congestive heart failure  Chronic obstructive pulmonary disease  Pulmonary fibrosis  Diabetes Mellitus  Previous Stroke  Chronic kidney disease  Hepatic Failure | 13  2  1  2  1  5  3  1  1 | 1  0  0  0  0  1  0  0  1 | 0.65  1.0  1.0  1.0  1.0  0.57  1.0  1.0  0.23 |
| Surgical Site  Right/Left | 26/10 | 4/1 | 0.31 |
| Baseline PaO2 | 76 (IQR 68 to 91) mmHg | 87 (IQR 74 to 92) mmHg | 0.16 |
| Baseline PaCO2 | 37 (IQR 35 to 42) mmHg | 39 (IQR 35 to 44) mmHg | 0.58 |
| Baseline FVC%*_a_* | 64 (SD ±22) | 55 (SD ±26) | 0.39 |
| Baseline FEV1%*_b_* | 64 (SD ±25) | 54 (SD ±24) | 0.40 |
| Baseline FEV1/FVC%*_c_* | 90 (IQR 62 to 120) | 103 (IQR 59 to 122) | 0.76 |
| Baseline maximal DIA*_d_* on operated side | 4.2 (SD ±1.4) mm | 3.2 (SD ±1.3) mm | 0.15 |
| Baseline maximal DIA*_d_* on non-operated side | 4.0 (SD ±1.3) mm | 3.8 (SD ±1.7) mm | 0.79 |
| Baseline maximal TF*_e_* on operated side | 53% (IQR 38 to 71) | 46% (IQR 31 to 62) | 0.33 |
| Baseline maximal TF*_e_* on non-operated side | 59% (IQR 40 to 75) | 43% (IQR 35 to 85) | 0.64 |

Abbreviation: *a*.FVC%, forced vital capacity percentage of predicted; *b*.FEV1%, forced expiratory volume in the first second percentage of predicted; *c*.FEV1/FVC% percentage of predicted ratio between FEV1 and FVC; *d*.DIA, diaphragmatic excursion; *e*.TF, thickening fraction.

Normally distributed variables are reported as mean and nonnormally distributed variables as median. Fisher exact test was used for dichotomous variables, t- student test for normally distributed continuous variables and non-parametric Mann-Whitney test for continuous variables not normally distributed.
